# mRNA fragments in *in vitro* culture media are associated with bovine preimplantation embryonic development

**DOI:** 10.3389/fgene.2015.00273

**Published:** 2015-08-24

**Authors:** Jenna Kropp, Hasan Khatib

**Affiliations:** Department of Animal Sciences, University of Wisconsin–Madison, MadisonWI, USA

**Keywords:** extracellular mRNA, embryo, media, IVF, small-RNA sequencing

## Abstract

*In vitro* production (IVP) systems have been used to bypass problems of fertilization and early embryonic development. However, embryos produced by IVP are commonly selected for implantation based on morphological assessment, which is not a strong indicator of establishment and maintenance of pregnancy. Thus, there is a need to identify additional indicators of embryonic developmental potential. Previous studies have identified microRNA expression in *in vitro* culture media to be indicative of embryo quality in both bovine and human embryos. Like microRNAs, mRNAs have been shown to be secreted from cells into the extracellular environment, but it is unknown whether or not these RNAs are secreted by embryos. Thus, the objective of the present study was to determine whether mRNAs are secreted into *in vitro* culture media and if their expression in the media is indicative of embryo quality. *In vitro* culture medium was generated and collected from both blastocyst and degenerate (those which fail to develop from the morula to blastocyst stage) embryos. Small-RNA sequencing revealed that many mRNA fragments were present in the culture media. A total of 17 mRNA fragments were differentially expressed between blastocyst and degenerate conditioned media. Differential expression was confirmed by quantitative real-time PCR for fragments of mRNA POSTN and VSNL-1, in four additional biological replicates of media. To better understand the mechanisms of mRNA secretion into the media, the expression of a predicted RNA binding protein of POSTN, PUM2, was knocked down using an antisense oligonucleotide gapmer. Supplementation of a PUM2 gapmer significantly reduced blastocyst development and decreased secretion of POSTN mRNA into the media. Overall, differential mRNA expression in the media was repeatable and sets the framework for future study of mRNA biomarkers in *in vitro* culture media to improve predictability of reproductive performance.

## Introduction

Extracellular RNA has become a focus for developing new diagnostic strategies for identifying disease or abnormal pathologies (Etheridge et al., 2013). Studies have shown that a class of small RNA, microRNAs (miRNAs), are secreted from tumor cells into the blood of patients and can be used to diagnose cancer with high specificity (Chen et al., 2008; Mitchell et al., 2008). miRNAs are relatively stable in the extracellular environment as they are either bound to proteins such as Argonaute 2 (Ago2) or are contained within exosomes (Valadi et al., 2007; Wang et al., 2010; Turchinovich et al., 2011). Likewise, mRNAs are packaged into exosomes and secreted by cells into the extracellular environment (Valadi et al., 2007). Neither the mechanism for selection of which RNAs are secreted, nor are the roles of extracellular miRNA and mRNA well understood. However, extracellular RNA may play a role in cell-cell communication (Valadi et al., 2007). While the function of extracellular RNA is largely unknown, the high stability and selective secretion into the environment allows for the potential to use these RNAs as biomarkers.

Recently, it has been demonstrated that miRNAs are selectively secreted from the developing embryo into the culture media and thus have strong potential as biomarkers of embryo development (Kropp et al., 2014; Rosenbluth et al., 2014). A previous study revealed 11 miRNAs to be highly expressed in the *in vitro* culture media of bovine growth-retarded embryos (deemed degenerate as they fail to develop from the morula to the blastocyst stage) in comparison to healthy blastocyst embryos (Kropp and Khatib, 2015). Similarly, miR-191 was found to be highly expressed in the culture media of chromosomally abnormal aneuploid embryos in comparison to euploid embryos (Rosenbluth et al., 2014). These studies have focused on the presence of miRNAs in *in vitro* culture media, but the secretion of mRNAs into the extracellular environment and their roles in embryonic development are poorly understood.

We hypothesize that, similar to other cell types, embryonic cells secrete mRNAs into *in vitro* culture media, which could provide a means to non-invasively survey the embryo for its developmental potential. To test this hypothesis, the media derived from embryos of differing competence or quality was deep-sequenced to characterize the RNA milieu. Many small mRNA fragments were identified and found to be differentially expressed in the media of embryos varying in developmental ability. Overall, these small mRNAs offer potential as non-invasive biomarkers of embryo development and fertility in cattle which could be translated into mammalian *in vitro* systems to improve reproductive efficiency.

## Materials and Methods

### Ethics Statement

This study is exempt from approval of the Institutional Animal Care and Use Committee because animals were not handled at our institution. Ovaries used for embryo production were purchased from Applied Reproductive Technology, LLC (Monona, WI, USA).

### *In Vitro* Production of Embryos and Media

Embryos and conditioned media were generated through an IVP system, as described by Kropp et al. (2014). In brief, ovaries were obtained from a local slaughterhouse and oocytes were aspirated from 2–8 mm follicles. Cumulus-oocyte complexes were washed twice in Vigro TL-Hepes (Bioniche, Pullman, WA, USA) and cohorts of 10 cumulus–oocyte complexes were then placed into a 50 μl drop of maturation medium. Following 24 h of incubation in maturation media, cumulus–oocyte complexes were removed from the media, washed once in supplemented TL-Hepes, and transferred to fresh 44 μl drops of IVF-TL medium (Millipore, Phillipsburg, NJ, USA). Sperm were prepared using a percoll gradient procedure (Parrish et al., 1995). The final concentration of sperm was adjusted to 1 million sperm/ml and added to each fertilization media droplet (designated day 0) at a volume of 2 μl along with 2 μl each PHE and heparin. Gametes were co-incubated for 24 h. Presumptive zygotes were then removed from fertilization medium, washed in supplemented TL-Hepes, and vortexed to strip away their cumulus cells. The presumptive zygotes were then transferred in cohorts of 25 to fresh 50 μl drops of culture medium supplemented with FAF-BSA, sodium pyruvate, amino acids, and gentamicin sulfate. Embryos produced for small-RNA sequencing were transferred into a commercial synthetic oviductal fluid (SOF) based culture medium (Millipore), whereas embryos produced in biological replicates to validate sequencing results were transferred into a CR_1aa_ based culture medium (Rosenkrans et al., 1993; Sagirkaya et al., 2006).

Embryos were cultured until day 5 of development and assessed morphologically for characteristics of a compacted morula described by Bo and Mapletoft (2013), as those in which the individual blastomeres are difficult to distinguish and have coalesced so that the embryo mass take up about 60–70% of the perivitelline space. Compacted morula were washed and transferred to a fresh drop of SOF or CR_1aa_ medium lacking FAF-BSA supplementation. These embryos were individually cultured until day 8 of development, at which point they were morphologically assessed. Embryos which failed to develop from the morula stage to the blastocyst stage within 72 h were deemed as degenerate and those which developed to the blastocysts stage with inner cell mass and trophectoderm were deemed as blastocysts. Media conditioned by mid- and expanded blastocysts of quality grades 1–2 (Bo and Mapletoft, 2013) were collected and pooled together. Likewise, media conditioned by degenerate embryos was collected and pooled together. Three IVP replicates were carried out for small-RNA sequencing and four additional IVP replicates were carried out to validate small-RNA sequencing expression (Supplementary Table S1). In each replicate, one pool of media was collected per embryo type; see Supplementary Table S1 for the pooling strategy. Additionally, a different sire was used for each replicate to eliminate a bull effect.

### RNA Extraction from Media

RNA was extracted from media samples using the miRNAeasy Serum/Plasma kit (Qiagen, Germantown, MD, USA) which is designed to isolate cell-free total RNA. Media samples destined for small-RNA sequencing were extracted three times with an initial input volume of 200 μl per sample to achieve input requirements for sequencing. Total RNA isolated following three extractions ranged from 730–1490 ng across the samples. Pools of media samples for validation were extracted once with an initial input of 200 μl of sample, and medium collected from a single embryo was extracted once with an initial volume of 50 μl.

### Library Preparation, Small-RNA Sequencing, and Data Analysis

Sample preparation and small-RNA sequencing procedures are described in detail in Kropp and Khatib (2015), but here are briefly summarized. The RNA libraries were prepared by LC Sciences (Houston, TX, USA) with equivalent amounts of RNA for each sample using the Illumina TruSeq^TM^ Small RNA Library Prep kit following the kit’s guidelines. The libraries were quality checked using an Agilent Bioanalyzer and then clustered on Illumina’s Cluster Station followed by sequencing on an Illumina GAIIx instrument. The raw sequenced reads were extracted, filtered and those which were between 15–32 nucleotides in length were clustered into families and deemed unique sequences (family of raw sequencing reads with similar sequences). Reads were then mapped to the bovine UMD_3.1 reference genome using Tophat, and Cuﬄinks was utilized for assembly. The total read fragments for each gene was then summed. Normalization of the reads and differential expression analysis were carried out in EdgeR using a negative binomial distribution to conduct a paired *t*-test. Significance level was set at *P* < 0.05.

### Validation of Differentially Expressed mRNAs by Quantitative Real-time PCR (qRT-PCR)

Subsets of significantly differentially expressed mRNA fragments in media were chosen to be validated both technically through qRT-PCR, and biologically in pools of media derived from four additional IVP experiments. The total RNA was extracted from each pool as described above with the addition of a spike-in control, *Caenorhabditis elegans* miR-39. The extracted RNA was then reverse transcribed into cDNA using a MiScript II RT kit (Qiagen). The HiFlex buffer was used for subsequent quantification of both mRNA and mature miRNA (the spike-in control). The cDNA reaction adds a universal tag sequence to the 5′ end which is necessary for subsequent steps to quantify these small fragments using the Qiagen PCR system. To assess gene expression, qRT-PCR was carried out using the miScript SYBR Green kit (Qiagen). Primer sequences are listed in Supplementary Table S2. The qRT-PCR reactions were carried in a Bio-Rad iCycler under the following conditions: 95°C for 15 min followed by 40 cycles of 94°C for 15 s, 55°C for 30 s, and 70°C for 30 s. Data were then analyzed using a paired *t*-test on the ΔCt values (Ct of mRNA – Ct of miR-39 spike-in control) for each mRNA fragment. The fold change in expression was calculated using the 2^-ΔΔCt^ method as described by Livak and Schmittgen (2001).

### Pumilio2 Antisense Oligonucleotide Gapmer Supplementation to Culture Media

To assess whether the RNA binding protein (RBP) of POSTN, Pumilio 2 (PUM2), affects the secretion of the fragment into the media, gene knockdown of *PUM2* in embryos was performed. Embryos were produced by IVP as described above; however, on day 5 of development a cohort of 5–15 morulae were transferred to fresh drop of CR1 culture media lacking FAF-BSA supplementation. Culture media containing the cohort of media was then either supplemented with an antisense oligonucleotide, gapmer LNA^TM^ longRNA GapmeR (Exiqon, Woburn, MA, USA), designed to target the RBP *PUM2* gene or water (vehicle of the antisense oligonucleotide). Cohorts of morulae were then cultured until day 8 of development and morphologically assessed. Embryos were deemed either a blastocyst or a degenerate, and the development rate was calculated as the percentage of morulae that developed to the blastocyst stage. Development was observed in three IVP replicates and blastocyst and degenerate embryos were collected, and pooled together for each treatment group. Media from supplemented and non-supplemented embryos were also collected for gene expression analysis of *POSTN*.

### Expression of POSTN mRNA Fragment in Media Following Antisense Oligonucleotide Supplementation

Following supplementation of the antisense gapmer to knockdown expression of *PUM2* within embryos, the expression of POSTN in the media was tested to determine if the RBP affects its secretion. Total RNA was extracted from media as aforementioned using the miRNeasy Serum/Plasma kit (Qiagen) where the initial input volume was 50 μl. A spike-in control, miR-39, was added at the time of extraction for downstream quantification of gene expression. Extracted RNA was reverse transcribed into cDNA using the miScript II RT kit (Qiagen) and qRT-PCR reactions were carried out using the miScript SYBR Green kit (Qiagen) as described above with primer sequences listed in Supplementary Table S3.

### Statistical Analysis

Statistical analysis of gene expression data and graph generation was conducted using the software OriginLab (OriginLab Corporation, Northampton, MA, USA). Expression data generated by qRT-PCR was analyzed using a paired *t*-test to assess the mean difference of the ΔCt between differing types of embryos and/or treatments for each IVP replicate. For each analysis, significance level was set at *P* < 0.05.

## Results

### Analysis of small-RNA Sequencing Data

Small-RNA sequencing data revealed 9,128,304 total reads mapped to mRNA across the media samples. Differential gene expression was estimated using the number of reads summed for each sample. A total of 17 genes were found to be differentially expressed between blastocyst and degenerate media, in which 13 genes were more highly expressed in blastocyst conditioned media and four were more highly expressed in degenerate conditioned media (**Table 1**). Interestingly, multiple mRNA fragments were identified for each gene; however, several demonstrated expression of a predominant fragment based on the number of normalized reads per sample.

**Table 1:** Differential expression of mRNA fragments in *in vitro* culture media conditioned by blastocyst or degenerate embryos.

Gene symbol	Predominant fragment sequence	Fold change (log2)
**Highly expressed in blastocyst media**		
VSNL1	TGCTTGGACTACATA	4.24
LOC784070	ACAACCTCAACTCCTA	5.86
POSTN	TGGTTGAGGGTTGTA	6.28
LOC504833	CCACCTCGTTCCCGTGG	3.92
RIC3	ATAATTTTGAACGAAA	4.37
KCTD12	CCACCACGTTCTCGTGT	5.52
NDST2	CCGCCACGTCCCGTGG	3.89
FSCN3	CGCTGTGGGATGAACCA	4.15
DLL4	CCCCCACCTTCCCGTGG	3.70
LOC785098	CACCACGTTCCCGTGT	2.45
DAGLB	TCCCGGCCGATGCACCA	2.83
GCLC	CCCGGGTGTCCCCTCCA	3.78
ABCC1	CACCACGTTCACGTGG	2.02
**Highly expressed in degenerate media**		
NETO2	AGAACTTGAAGGACCG	3.26
ZNF319	CGGCCCTGGCGGAGCGC	2.35
NAT12	CCTCCGTTGCCCTCAGCC	3.67
LOC783819	GCGGTCGGCGTCCCC	3.15

### Gene Expression Validation

**FIGURE 1:**
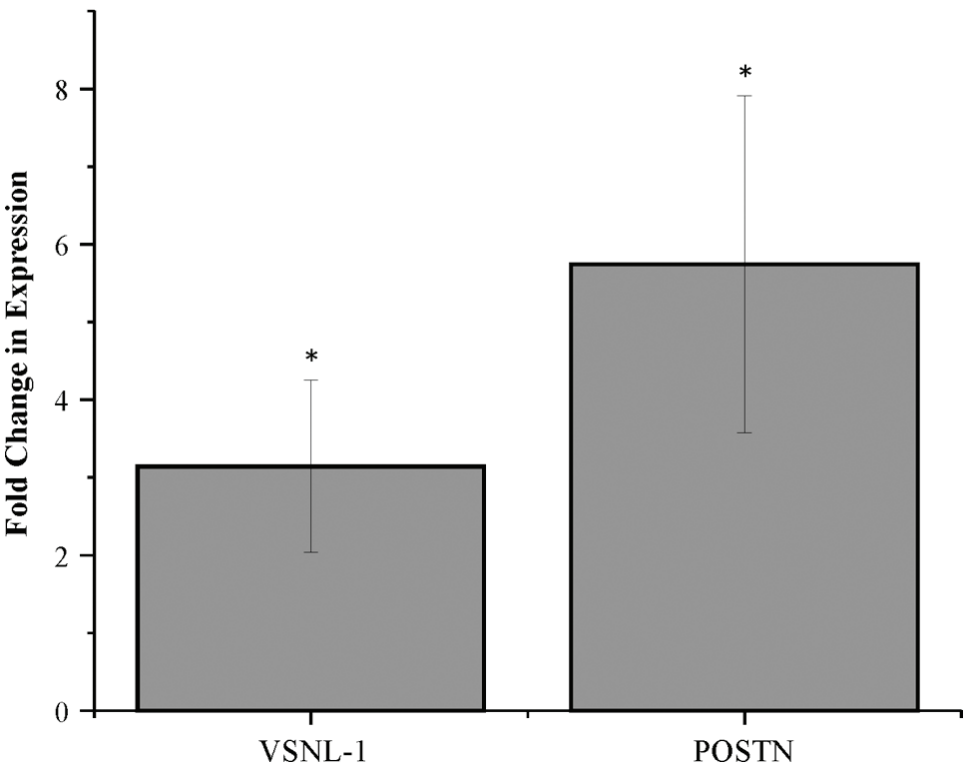
**Differential expression of mRNA fragments in blastocyst conditioned media compared to degenerate conditioned media.** Error bars represent the SE of the mean fold change in expression calculated across four biological replicates. ^∗^*P* < 0.05.

To confirm the differentially expressed mRNA fragments obtained by RNA sequencing, two mRNA fragments were further tested through qRT-PCR in media samples generated in four additional IVP experiments. Expression of small mRNA fragments mapped to the genes visinin-like 1 (*VSNL-1*) and periostin (*POSTN*; also known as osteoblast specific factor or OSF) was confirmed as more highly expressed in blastocyst conditioned media compared to degenerate conditioned media (**Figure 1**). The fold changes in expression for VSNL-1 and POSTN mRNA fragments were 3.14 ± 1.10 SE (*P* < 0.05) and 5.74 ± 2.16 SE (*P* < 0.05), respectively. Furthermore, to test whether mRNA fragments can be detected in the medium of single embryos, POSTN fragment expression was measured in media samples from individual blastocysts (*n* = 9) and degenerates (*n* = 9). The POSTN mRNA fragment was expressed in each individual media sample, though there was no significant difference in expression between the mean of the individual ΔCts calculated for blastocyst and degenerate conditioned media (data not shown).

### Supplementation of PUM2 Gapmer Decreases Embryo Development and Results in Reduced POSTN Expression in Media

Interestingly, the mRNA fragments identified in media all contain an RNA-binding protein motif within the short sequence. Using RBPDB (http://rbpdb.ccbr.utoronto.ca/index.php), the database of RNA-binding protein specificities, it was found that the POSTN fragment contained a sequence recognition motif for the RNA-binding protein Pumilio2 (PUM2). To assess the role of PUM2 on POSTN mRNA secretion, an antisense oligonucleotide, gapmer, was supplemented to the culture media of morula stage embryos. The gapmer is composed of DNA monomers complementary to a target mRNA sequence flanked by modified nucleotides called locked nucleic acids (LNA) which confers high stability to the gapmer (Kurreck et al., 2002; Kauppinen et al., 2005). Upon gapmer binding to its targeted mRNA, a heteroduplex will form and RNase H will be recruited to cleave the RNA target strand (Kurreck et al., 2002; Kauppinen et al., 2005).

Supplementation of a PUM2 gapmer to morulae stage embryos across three IVP replicates significantly reduced development to the blastocyst stage by 11.6% (*P* = 0.025; **Table 2**). Embryos were collected and the expression level of *PUM2* was quantified to determine the degree of expression knockdown. Quantification of *PUM2* mRNA expression in blastocysts that developed within supplemented media was not significantly different than control blastocysts. The unaltered expression level could be a result of primer design or dosage compensation of expression within embryos from day 5 to day 8 in development.

**Table 2:** Embryonic development following supplementation of a PUM2 GapmeR compared to control.

Treatment group	Total morula	Mean number of blastocysts (±SE)	Mean number of degenerates (±SE)	% Morula developed to blastocyst
Control 1 μM PUM2 GapmeR	98 93	21 ± 3.8 16.3 ± 3.8	11.7 ± 1.9 14.7 ± 2.6	64.3%^a^ 52.7%^b^

*Data represents the total number of morula and the mean number of blastocysts and degenerates ±SE across three IVP replicates. Differing superscripts within a column denote statistically significant differences (*P* < 0.05).*

To assess whether the level of POSTN secretion was altered following PUM2 gapmer supplementation, expression of the POSTN fragment was quantified in the media. The level of the POSTN mRNA fragment in gapmer supplemented media was reduced by 60% in comparison to controls. The mean fold change of control blastocysts to gapmer supplemented blastocysts was 2.67 ± SE 0.47 (*P* < 0.05). Overall, supplementation of a PUM2 gapmer to the media resulted in reduced embryonic development to the blastocyst stage and reduced level of the POSTN mRNA fragment secreted into the media.

## Discussion

We hypothesized that mRNAs are secreted into *in vitro* culture media and can be used as biomarkers of embryonic development. Evidence from small-RNA sequencing data of media demonstrates that mRNAs are secreted by embryos and are differentially expressed between embryos of differing developmental competence. Expression of POSTN and VSNL-1 mRNA fragments was higher in blastocyst media than degenerate media. These expression differences were first observed across three biological replicates that underwent small-RNA sequencing and further confirmed by qRT-PCR in four additional biological replicates. It can be concluded that small mRNA fragments are secreted into the culture media and are reproducible, hence setting the framework for future development of biomarkers reflecting embryo development and quality.

The mRNA fragments detected in this study were highly reproducible demonstrating preferential secretion from the embryo and stability within the extracellular environment. Several mRNA fragments mapped to the 3′UTR regions and many contained RBP sequence motifs. This finding is consistent with a study which identified mRNA fragments, within exosomes secreted by cultured human glioblastoma cells that were biased toward the 3′UTR using microarray analysis (Batagov and Kurochkin, 2013). Exosomal mRNA degradation and turnover is an intrinsic cellular process which occurs within both the nucleus and cytoplasm (Chlebowski et al., 2013). However, short fragment length may also be explained by the premise that the fragments mapped to the 3′UTR are protected by RBPs. Indeed, it has been reported that extracellular miRNAs are largely protein-bound whereas a smaller portion is contained within exosomes (Wang et al., 2010; Turchinovich et al., 2011). Studies have demonstrated that extracellular mRNAs are contained within vesicles such as microvesicles and exosomes; however, whether mRNAs are bound to proteins is unreported (Valadi et al., 2007). Within a cell, RBPs bind to the 3′UTR regions of transcripts whereby they act to regulate the localization of the transcript, translational repression or activation, and stability (Idler and Yan, 2012). It is plausible that these RBPs may serve to protect the fragments from degradation outside of the cell and play a role in localization to the extracellular environment.

Interestingly, the gene corresponding to the POSTN mRNA fragment identified in media has been previously identified to have a role in embryonic development and in mediating the uterine environment during pregnancy (Ahn et al., 2009; Morelli et al., 2014). In pregnant ewes, endometrial POSTN mRNA increases from days 12–14 in comparison to cyclic ewes, and the protein is detectable in uterine flushings of pregnant ewes but not in cyclic ewes (Ahn et al., 2009). Moreover, the study observed that treatment of recombinant human POSTN protein to *in vitro* cultured ovine trophectoderm cells mediated attachment to the culture wells coated with POSTN and stimulated migration (Ahn et al., 2009). Similarly, a study found that POSTN mRNA expression was high in trophoblastic and decidual samples of women undergoing voluntary pregnancy termination in comparison to those who had experienced spontaneous pregnancy loss (Morelli et al., 2014). Remarkably, western blot analysis of serum revealed that POSTN protein levels were high in women undergoing spontaneous pregnancy termination compared to women who had experienced spontaneous pregnancy loss; whereas POSTN was not detected in the serum on non-pregnant women (Morelli et al., 2014). These studies suggest that POSTN mRNA and protein expression in tissues and in uterine fluid play a role in embryo development and supports establishment of pregnancy. The present study identified POSTN fragment secreted by blastocyst embryos, however, future studies are needed to determine whether POSTN is involved in cell signaling at the maternal–fetal interface.

The mechanism of selection and secretion of mRNAs into culture media is unknown. In the present study, the POSTN fragment contained a RBP sequence motif for PUM2. To assess whether the RBP had an effect on secretion of the fragment, an antisense oligonucleotide gapmer which targets PUM2 was supplemented to the media of morula stage embryos. Successful uptake of gapmers from culture media without transfection reagents, a process deemed “gymnosis,” has shown to efficiently reduce gene expression of target mRNA in cell culture experiments (Stein et al., 2010; Soifer et al., 2012). Supplementation of a PUM2 gapmer to morula stage embryos resulted in decreased embryonic development, as more embryos degenerated from the morula stage and concomitantly the concentration of the POSTN mRNA fragment was reduced in the media. Thus, supplementation of the gapmer identified PUM2 as a candidate gene associated with bovine embryonic development. Previous studies have demonstrated that PUM proteins have a role in regulating gene expression in the germline, embryonic development, and stem cell renewal (Asaoka-Taguchi et al., 1999; Spassov and Jurecic, 2003; Zhang et al., 2015). PUM1 and PUM2 are proteins within the highly conserved PUF family of RBS, which have been shown to repress gene expression (Van Etten et al., 2012). A study analyzing potential mRNA targets of PUM proteins found that about 15% of the cell’s transcriptome is potentially regulated by PUM proteins and about 74% of PUM2 targets contain a consensus motif in their 3′UTRs (Galgano et al., 2008). Furthermore, the consensus sequences within the 3′UTRs are in close proximity to predicted miRNA binding sites suggesting there may be interplay in gene regulation by RBPs and miRNAs (Galgano et al., 2008). A better understanding of the complex gene regulatory network by both RBPs and miRNAs on target mRNA and their effect on embryonic development is needed. Additionally, future studies should determine whether these RBPs are directly associated with secretion of mRNA fragments.

The antisense gapmer was supplemented to morula to reduce the expression of the RBP PUM2; however, by day 8 of development no difference in PUM2 expression was observed in control and supplemented blastocyst embryos. Given that the gapmer was supplemented at the morula stage, it is possible that expression was reduced at this stage; however, expression was measured at the blastocyst stage allowing ample time to recover from repressed mRNA expression as the embryo developed from the morula to blastocyst stage. Lack of gene expression differences could be due to dosage compensation of the embryo following supplementation or that the level of mRNA does not reflect changes in the protein being produced. Another explanation is poor primer specificity for the PUM2 transcripts being targeted. Several primers were tested for many transcript variants of PUM2; however, the transcript was not detected or no expression differences were observed. Better annotation of the bovine genome would allow assessment of each transcript variant and warrants future characterization of each variant’s expression in embryos.

The role of mRNAs in the extracellular environment has yet to be fully elucidated, however, it has been proposed that transfer of mRNAs through extracellular vesicles such as microvesicles and exosomes may play a role in cell-cell communication (Valadi et al., 2007). Several studies have identified mRNAs present in microvesicles and exosomes secreted into the extracellular space including blood serum and cell culture media (O’Driscoll et al., 2005; Ratajczak et al., 2006; Valadi et al., 2007; Zhang et al., 2012). Functionally, few studies have demonstrated uptake of exosomes and microvesicles by recipient cells where mRNA was not only delivered but also translated into protein (Ratajczak et al., 2006; Valadi et al., 2007; Skog et al., 2008). Thus, it could be hypothesized that mRNAs present in culture media may play a functional role in signaling to uterine epithelial cells and further studies are warranted.

## Conclusion

The present study identified a large population of mRNA fragments within the *in vitro* culture medium conditioned by embryos. It is plausible that the extracellular mRNAs may play a role in signaling at the maternal–fetal interface; however, further studies are warranted. Across several IVP replicates, the POSTN fragment was more highly expressed in blastocyst media in comparison to degenerate media thus providing a molecular biomarker to non-invasively survey embryonic development. Future studies should focus on developing a sensitive assay to survey single embryos and to determine if the biomarker of development correlates with pregnancy outcome.

## Author Contributions

JK participated in the design of the study and carried out the experiments, data analysis, and drafting of the manuscript. HK conceived the study and participated in the design of experiments and drafting of the manuscript.

## Conflict of Interest Statement

The authors declare that the research was conducted in the absence of any commercial or financial relationships that could be construed as a potential conflict of interest.
