# Supplementary material for: mRNA fragments in in vitro culture media are associated with bovine preimplantation embryonic development
Source: Front Genet. 2015 Aug 24;6:273. doi: 10.3389/fgene.2015.00273 (PMC4547040; doi:10.3389/fgene.2015.00273)
Supplement: Supplementary file 1 [file Table_1.DOCX]

**Supplementary Table 1.** IVP Production and Media Pooling Strategy. Replicates represent an IVP experiment in which a separate bull was used to fertilize a population of oocytes to generate media pools for RNA-Seq (n= 3 IVP replicates) and expression validation (n=4 IVP replicates. Pools were constructed for each replicate by combining 50 µl media drops of culture media for each embryo type to represent a blastocyst (B) and a degenerate (D) conditioned media pool.

| **Replicate** | **Bull ID** | **Total Oocytes** | **Number of Morula** | **Media Drops**  **(n) per pool** | |
| --- | --- | --- | --- | --- | --- |
|  |  |  |  | **B** | **D** |
| **RNA-Seq Replicates** | | | | | |
| 1 | A | 574 | 51 | 31 | 25 |
| 2 | B | 398 | 36 | 11 | 25 |
| 3 | C | 647 | 87 | 27 | 60 |
| **Expression Validation Replicates** | | | | | |
| 1 | A | 190 | 24 | 15 | 9 |
| 2 | B | 167 | 21 | 12 | 9 |
| 3 | C | 278 | 33 | 15 | 18 |
| 4 | D | 241 | 29 | 19 | 10 |
