# Supplementary material for: mRNA fragments in in vitro culture media are associated with bovine preimplantation embryonic development
Source: Front Genet. 2015 Aug 24;6:273. doi: 10.3389/fgene.2015.00273 (PMC4547040; doi:10.3389/fgene.2015.00273)
Supplement: Supplementary file 2 [file Table_2.DOCX]

**Supplementary Table 2. Primer Sequences for qRT-PCR validation of mRNA fragments**

| Gene | Primer Sequence  (5’–3’) |
| --- | --- |
| VSNL-1 | UGCUUGGACUACAUAUG |
| POSTN | UUGGUUGAGGGUUGUA |
| miR-39* | UCACCGGGUGUAAAUCAGCUUG |

* *C. elegans* miR-39 Spike-in Control
