# Supplementary material for: mRNA fragments in in vitro culture media are associated with bovine preimplantation embryonic development
Source: Front Genet. 2015 Aug 24;6:273. doi: 10.3389/fgene.2015.00273 (PMC4547040; doi:10.3389/fgene.2015.00273)
Supplement: Supplementary file 3 [file Table_3.DOCX]

**Supplementary Table 3.** Primers sequences for qRT-PCR of full length mRNA transcripts

| **Gene** | **Primer Sequence**  **(5’–3’)** | **Amplicon Size**  **(bp)** |
| --- | --- | --- |
| *PUM2* | F: TGTCTCCGCGATCAGAAAGT | 124 |
|  | R: TCAGCATCTCTAGTGCCAAAAG |  |
| *GAPDH** | F: TGCCCAGAATATCATCCC | 134 |
|  | R: AGGTCAGATCCACAACAG |  |

*Reference gene
